# Stented Biological Prosthesis Versus Mitral Allograft in Surgical Treatment of Tricuspid Valve Infective Endocarditis

**DOI:** 10.31083/RCM37204

**Published:** 2025-07-08

**Authors:** Mikhail Nuzhdin, Yury Malinovsky, Maksim Galchenko, Roman Komarov, Aleksey Fokin, Nikita Nadtochiy

**Affiliations:** ^1^Department of Cardiac Surgery, State Budgetary Healthcare Institution “Chelyabinsk Regional Clinical Hospital”, 454048 Chelyabinsk, Russian Federation; ^2^Department of Surgery, South Ural State Medical University, 454092 Chelyabinsk, Russian Federation; ^3^Department of Applied Sciences and Data Analysis, Saint-Petersburg State Agrarian University, 196605 Saint-Petersburg, Russian Federation; ^4^Department of Aortic and Cardio-Vascular Surgery, I.M. Sechenov First Moscow State Medical University, 119146 Moscow, Russian Federation

**Keywords:** endocarditis, allograft, tricuspid valve replacement

## Abstract

**Background:**

The prevalence of tricuspid valve (TV) infective endocarditis (IE) continues to increase among patients with drug addictions and chronic vascular access or cardiac electronic devices. Moreover, long-term mortality and morbidity following surgery with conventional prostheses remain high. Allografts may represent a suitable alternative in tricuspid surgery. This study aimed to compare outcomes between stented biological valves and mitral allografts (MAs) for tricuspid valve replacement (TVR).

**Methods:**

A total of 54 patients with IE underwent TVR using either a stented bioprosthesis (B) or MA between January 2016 and July 2024. Clinical and echocardiographic data were analyzed in accordance with the Tricuspid-Valve Academic Research Consortium (T-VARC) criteria. Early safety, clinical efficacy, and time-to-event survival were compared between the two equal B and MA groups.

**Results:**

There were no in-hospital or 30-day mortality, nor cardiac, cerebral, and wound complications in either group. The peak and mean pressure gradient (PG) on TV after surgery were 9.2 (6.5–12.0) and 4.0 (3.2–6.0) mmHg in the B group versus 6.0 (4.5–7.5) and 3.0 (2.0–4.0) mmHg in the MA group (*p* < 0.001). A T-VARC-adjusted analysis demonstrated superior freedom from cardiovascular mortality, recurrent IE, reoperation, and permanent pacemaker implantation (PPI) in the MA group 2 years after operation. Kaplan–Meier analysis revealed significantly higher freedom from cardiovascular mortality in the MA group (100% vs. 81.5%, 77.8%, 77.8%, 69.6% respectively (log-rank test, *p* = 0.011) at 12-, 18-, 24-, 36-months, and freedom from PPI (100% vs. 81% at all time intervals) (log-rank test, *p* = 0.021).

**Conclusion:**

Application of contemporary endpoint criteria demonstrated superior outcomes with MA, including lower cardiovascular mortality, reduced PPI, fewer recurrent endocarditis, decreased reoperations, cardiac hospitalizations, alongside improved patient-reported outcomes. Time-to-event analysis demonstrated benefits in cardiovascular survival and PPI avoidance with allografts. Mitral allograft may be a preferable alternative valve substitute for TVR in patients with IE.

**Clinical Trial Registration:**

ClinicalTrials.gov ID: NCT06591000, https://clinicaltrials.gov/study/NCT06591000?term=NCT06591000&rank=1, registration date: September 19, 2024.

## 1. Introduction

Tricuspid valve infective endocarditis (TV IE) accounts for up to 10% of all cases of IE, with most cases being associated with intravenous drug use [1]. Appropriate treatment of TV IE is critical in contemporary practice, particularly given the increasing number of patients requiring intracardiac electronic devices [2]. Advances in preoperative management and patient selection have reduced complication rates during tricuspid valve replacement (TVR), yielding promising long-term outcomes [3, 4]. Conventional surgical approaches for TV IE include TVR with mechanical or biological prostheses, valve repair, or valvectomy [5]. However, these methods possess significant disadvantages. For example, the mechanical valves necessitate lifelong anticoagulation and pose thromboembolic risks. In comparison, bioprostheses are prone to degeneration and recurrent endocarditis [6]. Although allograft valves have not been widely adopted in clinical practice, emerging evidence supports their efficacy in selected patient cohorts [7, 8, 9].

However, inconclusive data exist regarding reporting outcomes after TV surgery, even in recently published studies [5, 9]. This retrospective single-center study used contemporary endpoint definitions to compare two valve substitutes in the surgical management of TV IE regarding their safety and clinical efficacy.

## 2. Materials and Methods

### 2.1 Study Population and Data Source

The study evaluated early safety and clinical efficacy in patients who underwent surgical treatment for TV IE at a tertiary referral center between January 2016 and July 2024. This study was approved by the South Ural State Medical University and Chelyabinsk Regional Clinical Hospital institutional review board (protocol 3/14/01/2016). Further, this study complied with the following provisions and principles: the ALLOTRI clinical study (ClinicalTrials.gov ID: NCT06591000); the ethical standards of the responsible institution on human subjects; the Helsinki Declaration. Written informed consent was obtained from all patients before surgery. Clinical data were obtained from the electronic medical database and patient case notes. The surgical procedure was decided according to recent guidelines for treating IE. All preoperative and postoperative diagnostic investigations were performed at the same diagnostic department. Postoperative follow-up was conducted exclusively at the outpatient department at our institution to ensure consistency.

### 2.2 Inclusion Criteria

(1) Primary TV pathology or bioprosthetic valve failure (endocarditis) requiring surgical intervention.

(2) Preoperative/intraoperative assessment indicating TVR as the preferred approach over repair.

(3) Complete medical records in the regional database, including follow-up contact information.

### 2.3 Exclusion Criteria

(1) Pregnancy.

(2) Confirmed active drug addiction.

(3) Progressive HIV infection.

(4) HIV-infected patients (CD4 <250 cells/mm^3^).

(5) Secondary tricuspid valve pathology (left-sided valve disease).

(6) Left ventricle ejection fraction (LVEF) <40%.

(7) Age under 18 and more than 70 years.

### 2.4 Endpoint Definitions

Efficacy endpoints included clinical, patient-centered, and surrogate outcomes adopted from the Tricuspid-Valve Academic Research Consortium (T-VARC) expert review [10].

#### 2.4.1 Definition of Early Safety (30-Day Postoperative Assessment)

Absence of procedural mortality or stroke, adequate performance (absence of tricuspid stenosis, mean gradient <5 mmHg, reduction in total tricuspid regurgitation to trivial or mild, freedom from unplanned surgical or interventional procedures related to the prosthesis, absence of life-threatening bleeding (T-VARC 5), no major cardiac structural complications, stage 2 or 3 acute kidney injury, myocardial infarction, any valve-related dysfunction or other complication requiring surgery or repeat intervention) [10].

#### 2.4.2 Clinical Efficacy (Annual Assessment) is Defined as a Composite of the Following

Freedom from all-cause and cardiovascular mortality, no rehospitalizations or reinterventions for tricuspid regurgitation/stenosis, heart failure, endocarditis relapse/recurrence, pacemaker implantation and improvement from baseline in symptoms (New York Heart Association (NYHA) improvement by more than 1 functional class); and/or improvement from baseline in quality-of-life (Kansas City Cardiomyopathy Questionnaire improvement) (KCCQ) by more than 5 points [10].

### 2.5 Infective Endocarditis Definition

Diagnostic assessment of IE complied with diagnostic criteria specified in the recent guidelines [11].

### 2.6 Surgical Technique and Valve Substitutes

All procedures were performed via median sternotomy, standard cannulation technique, and antegrade cold blood cardioplegia. Intraoperative transesophageal echocardiography was routinely performed in all cases. Implantation of stented biological valves was performed in standard fashion, using interrupted sutures with preservation of a part of the septal leaflet, provided the leaflet was not affected by endocarditis. The surgical mitral allograft (MA) implantation technique included several previously described steps and measures [12, 13]. The following stented porcine and bovine biological prosthesis were used for TVR: Edwards Lifesciences Perimount Magna (Edwards Lifesciences, Irvine, CA, USA), St’Jude Epic (St. Jude Medical, St. Paul, MN, USA), St’Jude Biocor (St. Jude Medical, St. Paul, MN, USA), and NeoCor (NeoCor Company, Kemerovo, Russian Federation). The valve sizes were selected following direct measurements, but varied from 27 (29) to 32 (33), depending on the manufacturer’s size. The median and interquartile range (IQR) bioprosthesis size was 31 (29–31).

All homografts were ordered from Saint-Petersburg Homograft Bank. The preservation and storage methods have been described previously [13]. Without formal sizing guidelines, allograft selection was guided by the preoperatively measured tricuspid annulus diameter and surgical experience. The criteria are the patient’s body size, right ventricle dimensions, and pulmonary artery pressure. Allografts were contraindicated in cases of severe pulmonary hypertension (≥40 mmHg) with concomitant right ventricular dilation due to potential adverse effects on outcomes. Oversized allografts (34–36 mm) were preferentially used for annuli >40 mm, leveraging the pliability of the tissue for adaptation to smaller annuli when needed. The median (IQR) MA size was 32 (30–32).

### 2.7 Data Analysis

All analyses were performed using Jamovi software (version 2.6, the Jamovi Project (The jamovi project (2025). jamovi (Version 2.6) [Computer Software]. Retrieved from https://www.jamovi.org)) and R package software (version 4.4 R Core Team (2024). R: A Language and environment for statistical computing. (Version 4.4) [Computer software]. Retrieved from https://cran.r-project.org. (R packages retrieved from CRAN snapshot 2024-08-07)). The dependent variables (before and after surgery, within one group of patients) were compared using the Wilcoxon test. The comparison between groups was performed using non-parametric statistics. Categorical variables are summarized as frequencies and percentages; comparisons were made using Fisher’s exact test (Haldane–Anscombe amendment was applied in cases with zero variables). A proportion difference test was used with Fisher’s exact test to compare early safety and clinical efficacy at exact time points. Continuous variables, expressed as the median with interquartile range, were compared using the Mann–Whitney *U* test. The Brunner–Munzel and median tests were also applied to determine statistically significant differences retrieved from the Mann–Whitney *U* tests. Kaplan–Meier curves were used to plot the survival and freedom from adverse events. Log-rank tests were used to compare the differences in the time-related outcomes between groups.

## 3. Results

### 3.1 Patients’ Characteristics

A total of 54 consecutive adult patients underwent surgery for TV IE between January 2016 and July 2024, after screening against inclusion/exclusion protocols. All patients met the IE diagnostic criteria for definite IE. Indications for surgery were based on the severity of heart failure symptoms, uncontrolled infectious process, vegetation size (>20 mm), or their combination. All patients were divided into two equal groups. MA has been predominantly utilized since 2021.

The preoperative data (Table 1) present minor differences in the body mass index (BMI) of patients, which was slightly lower in patients who underwent bioprosthesis implantation. However, the calculated medians and IQR in both groups were related to the normal BMI range. A higher prevalence of hepatitis C was noted in the stented bioprosthesis group. Assuming normal and comparable values of preoperative laboratory tests, functional status, and predicted mortality, assessed by score calculators, both groups were considered clinically comparable following further evaluation. The Kansas City Cardiomyopathy Questionnaire (KCCQ)12 test assessed self-reported quality of life. No preoperative differences were found with regard to this test, and the overall summary score reflected the NYHA III functional class.

**Table 1.** **Baseline patients’ characteristics**.

		Bioprosthesis	Allograft	*p*-value
Total N (%)		27 (50.0)	27 (50.0)	
Age, years		35.0 (29.0–39.5)	37.0 (33.0–42.5)	0.132
BMI, kg/m^2^		20.7 (20.1–22.8)	24.2 (21.1–26.6)	0.008
BSA, m^2^		1.7 (1.7–1.9)	1.9 (1.7–2.0)	0.063
EuroScore II		2.3 (1.5–3.5)	1.7 (1.4–2.8)	0.279
MELD/SMELD score		8.0 (7.0–9.0)	7.0 (7.0 –8.0)	0.080
Estimated 3-month mortality, % calculated				
	1.9%	17 (63.0)	24 (88.9)	0.054
	6%	10 (37.0)	3 (11.1)	
Tri-Score (predicted), points		4.0 (4.0–4.5)	4.0 (4.0–5.5)	0.703
Tri-Score predicted in-hospital mortality, %		8.0 (8.0–11.0)	8.0 (8.0–18.0)	0.703
Total bilirubin, µmol/L		11.1 (8.2–16.4)	12.5 (6.9–17.3)	0.723
Total protein, g/L		75.0 (68.5–79.0)	76.0 (74.2–81.6)	0.149
Creatinine level, µmol/L		82.0 (70.5–89.0)	84.0 (70.0–94.0)	0.822
Daily furosemide dose, mg/day, n (%)				
	20 mg	4 (14.8)	2 (7.4)	0.102
	40 mg	20 (74.1)	15 (55.6)	
	80 mg	3 (11.1)	9 (33.3)	
	120 mg	0 (0.0)	1 (3.7)	
Ascites, n (%)		1 (3.7)	3 (11.1)	0.610
Active IE, n (%)		12 (44.4)	11 (40.7)	1.000
NYHA, class				
	NYHA 2, n (%)	1 (3.7)	5 (18.5)	0.304
	NYHA 3, n (%)	21 (77.8)	18 (66.7)	
	NYHA 4, n (%)	5 (18.5)	4 (14.8)	
Pneumonia (septic embolic syndrome), n (%)		8 (29.6)	10 (37.0)	0.773
Positive blood culture, n (%)		13 (48.1)	8 (29.6)	0.264
Ongoing antibiotic treatment, n (%)		16 (59.3)	12 (44.4)	0.414
Bioprosthesis endocarditis, n (%)		6 (22.2)	5 (18.5)	1.000
Urgency				
	Elective, n (%)	10 (37.0)	12 (44.4)	0.925
	Emergent, n (%)	3 (11.1)	3 (11.1)	
	Urgent, n (%)	14 (51.9)	12 (44.4)	
Time from previous operation (months)		16.0 (10.0–19.8)	72.0 (69.0–84.0)	0.144
Hepatitis С, n (%)		25 (92.6)	17 (63.0)	0.019
HIV, n (%)		6 (22.2)	8 (29.6)	0.757
ARVT, n (%)		6 (22.2)	7 (25.9)	1.000
History of IVDA, n (%)		16 (59.3)	13 (48.1)	0.586
eGFR, mL/min/1.73 m^2^		107.6 (88.3–117.1)	99.9 (90.4–113.9)	0.511
FEV1, %		79.0 (72.5–89.0)	81.0 (71.0–89.0)	0.815
Baseline HGB, g/L		121.0 (96.0–143.0)	125.0 (112–133.5)	0.986
KCCQ12 summary score		44.8 (29.7–50.8)	36.5 (29.2–48.4)	0.396

Table footnote: Data are expressed as a number (n, (%)) or median (interquartile range). BMI, body mass index; BSA, body surface area; EuroScore II, European System for Cardiac Operative Risk Evaluation; MELD/SMELD, model for end-stage liver disease; Tri-Score, risk score model for tricuspid surgery; NYHA, New York Heart Association; HIV, human immune virus; ARVT, anti-retrovirus therapy; IVDA, intravenous drug abuse; eGFR, estimated glomerular filtration rate; IE, infective endocarditis; AV-block, atrioventricular block; AFib, atrial fibrillation; FEV1, forced expiratory volume in one second; HGB, hemoglobin; KCCQ, Kansas City Cardiomyopathy Questionnaire.

### 3.2 Operative Details and Early Outcomes

There were no in-hospital or 30-day mortality, acute myocardial infarction, stroke, or wound complications in either group. All patients demonstrated an uneventful early postoperative course, except for one case of postoperative bleeding, requiring resternotomy in the B group. Two patients (7.4%) underwent permanent pacemaker implantation (PPI) for complete atrioventricular block (AV block) within 14 days after bioprosthetic TVR. Although allograft implantation is a more time-consuming procedure, this implantation was not associated with longer ventilation time, increased blood transfusion requirements, drainage losses, or other complications. Notably, the allograft group required significantly fewer fresh frozen plasma transfusions (**Supplementary Table 1**), likely reflecting evolving transfusion protocols in the department (**Supplementary Table 1**).

### 3.3 Echocardiographic Data

Preoperative and postoperative echocardiographic assessments were performed at the same diagnostic department. All parameters reflected chronic right heart overload secondary to TV regurgitation (**Supplementary Table 2**). The dimensions of the left chambers remained within normal ranges, except for the left atrial volume index, which showed minor intergroup differences. Given the median values of this parameter in both groups, these differences appear clinically insignificant and likely represent confounding variation. MA replacement resulted in significantly lower pressure gradients on the TV and greater reductions in pulmonary systolic pressure after surgery, confirmed by the Mann–Whitney *U* and Brunner–Munzel tests (**Supplementary Table 2**).

### 3.4 Early Safety

Early safety was summarized after 30 days of surgery (during the first-year follow-up) in all patients and is presented in Table 2.

**Table 2.** **Early safety**.

	Bioprosthesis	Allograft	Fisher’s exact test	Proportion’s difference test		
	(n = 27)	(n = 27)	*p*-value	OR	95% CI	*p*-value (two-tailed)
Early cardiovascular mortality, n (%)	6 (22%)	0	0.023	16.63	0.89–311.79	0.017
Early prosthesis-related mortality, n (%)	6 (22%)	0	0.023	16.63	0.89–311.79	0.017
Early non-cardiovascular mortality, n (%)	2 (7.4%)	2 (7.4%)	1.000	1.00	0.16–6.28	1.000
PPI, n (%)	4 (14.8%)	0	0.111	0.09	0.005–1.857	0.077
AKI 2–3, n (%)	5 (18.5%)	0	0.051	0.07	0.004–1.419	0.037
Mean TV PG >5 mmHg, n (%)	9 (33.3%)	1 (3.7%)	0.011	0.11	0.02–0.68	0.006

Table footnote: PPI, permanent pacemaker implantation; AKI, acute kidney injury; TV PG, tricuspid valve pressure gradient; OR, odds ratio; CI, confidence interval.

#### 3.4.1 Mortality

Early mortality included cardiovascular, prosthesis-related, and non-cardiovascular causes. A significant intergroup difference was observed in cardiovascular mortality, primarily related to prosthesis failure (endocarditis) in the bioprosthesis group during the first postoperative year. Early non-cardiovascular mortality was identical in both groups (two patients), and comprised accidental trauma and a car accident in the bioprosthesis group versus intentional self-harm and throat cancer in the allograft group.

#### 3.4.2 Pacemaker Implantation

Four patients in the bioprosthesis group required PPI (14.8%), including two early cases. Meanwhile, no statistical difference was found between groups during the early safety analysis.

#### 3.4.3 Kidney Injury

Acute kidney injury (AKI) was defined according to the T-VARC consensus. At least stage 2 AKI developed in five (18.5%) patients in the bioprosthesis group (*p* = 0.051). The proportion difference analysis confirmed statistical significance (two-tailed *p* = 0.037); no patients required hemodialysis.

#### 3.4.4 Hemodynamic Valve Performance

T-VARC-defined adequate valve performance (no stenosis, mean gradient <5 mmHg) was not achieved in one-third of patients in the bioprosthesis group (9; 33.3%) despite a large bioprosthesis size (29–33 mm). Only the allograft recipient did not meet the proper gradient for TV; this difference was statistically significant (Fisher’s exact test and proportion difference analysis). Optimal/acceptable tricuspid regurgitation reduction was achieved universally.

### 3.5 Clinical Efficacy

Postoperative clinical efficacy was assessed at the 2-year follow-up (Table 3). All-cause mortality remained statistically unchanged from those data derived from the 1-year follow-up (early safety), with one additional non-cardiac death (drug overdose) in the MA group. Neither all-cause mortality nor freedom from reoperation reached statistical significance between groups. All other T-VARC endpoints demonstrated statistically significant differences between groups 2 years after surgery. A total of 12 patients in the B group were free from cardiovascular hospitalization (48%), with 22 (81.5%) in the MA group (Fisher’s exact test, *p* = 0.019). A total of 13 patients in the B group achieved an improvement of more than 5 points in the KCCQ (56.5%), and 25 (92.6%) patients from the MA group (Fisher’s exact test, *p* = 0.006).

**Table 3.** **Two years of clinical efficacy**.

	Bioprosthesis	Allograft	Fisher’s exact test	Proportion’s difference test		
	(n = 27)	(n = 27)	*p*-value	OR	95% CI	*p*-value (two-tailed)
All-cause mortality, n (%)	8 (29.6%)	3 (11.1%)	0.175	0.33	0.83–1.30	0.108
Freedom from IE, n (%)	18 (66.7%)	25 (92.6%)	0.039	0.19	0.04–0.87	0.021
Freedom from clinically significant valve dysfunction, n (%)	18 (66.7%)	26 (96.3%)	0.011	0.11	0.02–0.68	0.006
Freedom from mean TV PG >5 mmHg, n (%)	12 (44.4%)	26 (96.3%)	<0.001	0.05	0.01–0.28	<0.001
Freedom from TV vc >7 mm, n (%)	15 (55.6%)	24 (88.9%)	0.014	0.18	0.05–0.68	0.006
Freedom from PPI, n (%)	22 (81.5%)	27 (100%)	0.051	0.07	0.004–1.419	0.037
Freedom from reoperation, n (%)	21 (77.8%)	26 (96.3%)	0.100	0.19	0.03–1.21	0.062
NYHA improvement, n (%)	13 (48.1%)	25 (92.6%)	<0.001	14.48	2.34–89.82	<0.001

Table footnote: IE, infective endocarditis; TV vc, tricuspid valve vena contracta.

### 3.6 Longitudinal Study and Time-To-Event Survival

#### 3.6.1 All-Cause Mortality

**Fig. 1.**
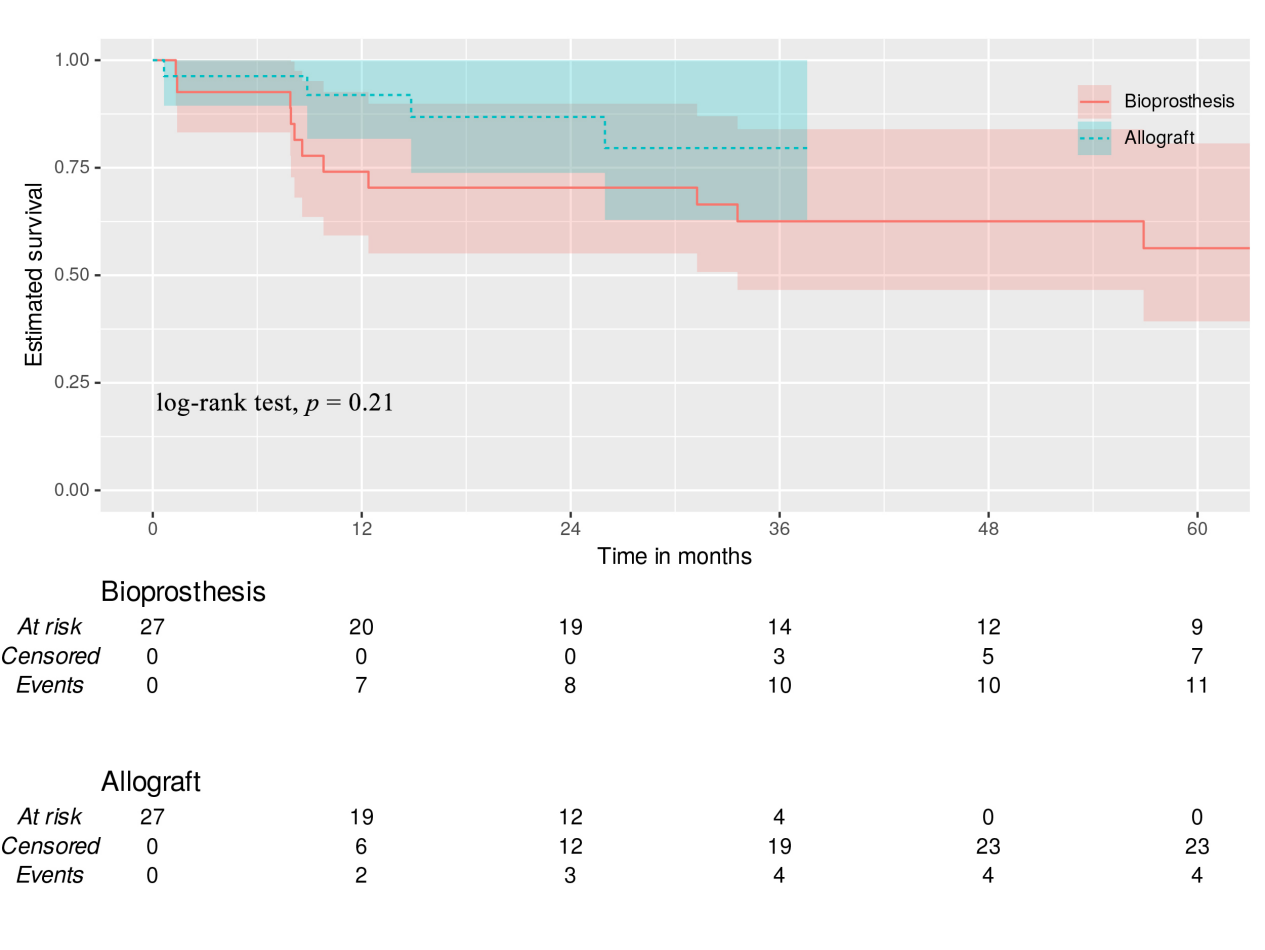
**All-cause mortality**. Green dotted line—allograft with 95% CI. Red line—bioprosthesis with 95% CI.

Time-to-event survival was estimated postoperatively at 12, 18, 24, and 36 months. No statistically significant difference was observed in all-cause mortality between patients who underwent bioprosthesis and MA (74.1%, 70.4%, 70.4%, 62.6% vs. 91.9%, 86.8%, 86.8%, 79.6%, respectively; log-rank test, *p* = 0.21) (Fig. 1).

#### 3.6.2 Cardiovascular Mortality

**Fig. 2.**
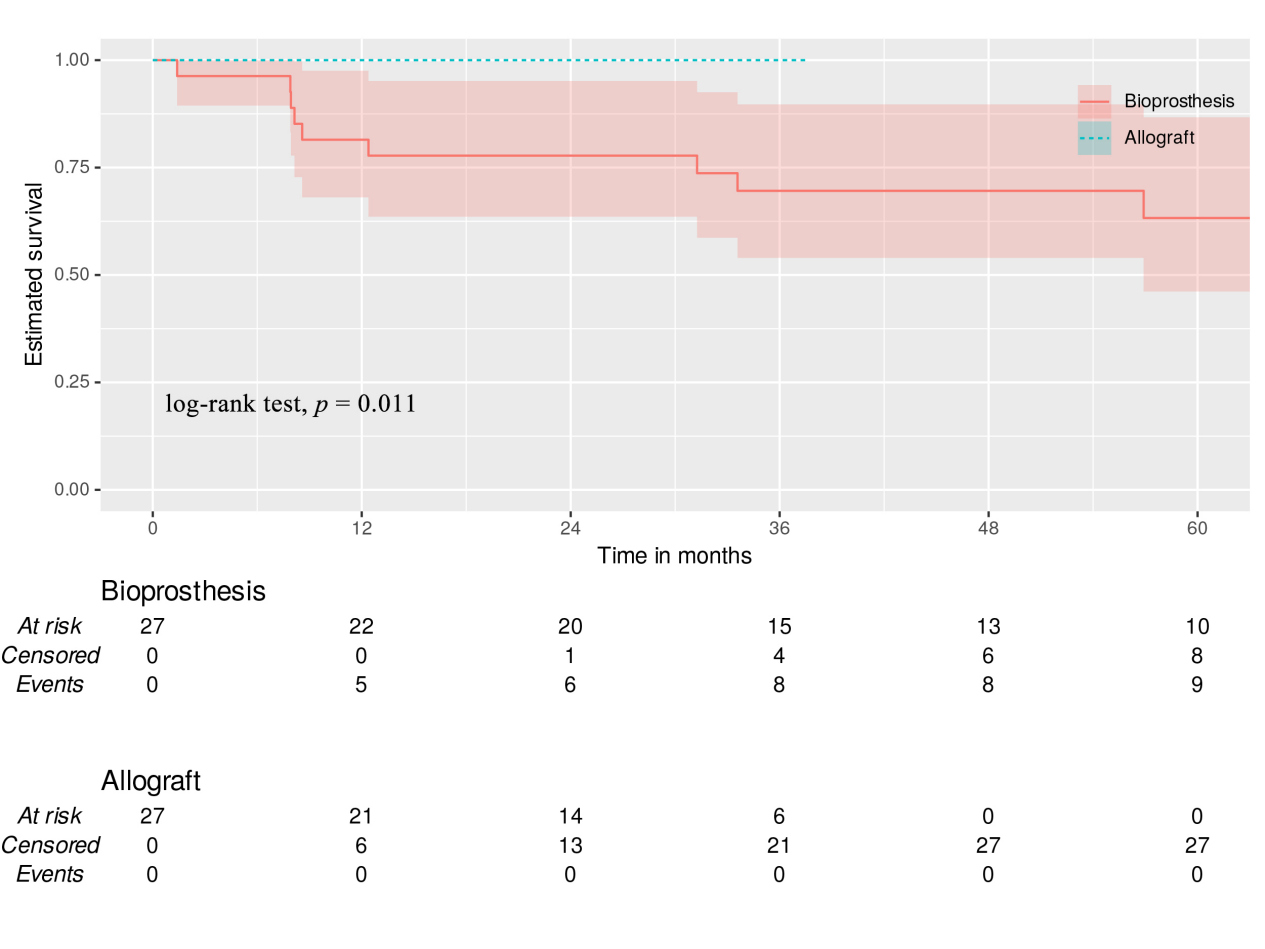
**Cardiovascular mortality**. Green dotted line—allograft with 95% CI. Red line—bioprosthesis with 95% CI.

Allograft implantation demonstrated significantly superior freedom from cardiovascular mortality at 12-, 18-, 24-, and 36-months post-operation compared to bioprosthesis TVR (100% vs. 81.5%, 77.8%, 77.8%, 69.6% respectively; log-rank test, *p* = 0.011; Fig. 2). All cardiovascular deaths in the bioprosthesis group were device-related (acute valve thrombosis, septic shock, multi organ failure).

#### 3.6.3 Endocarditis Recurrence

**Fig. 3.**
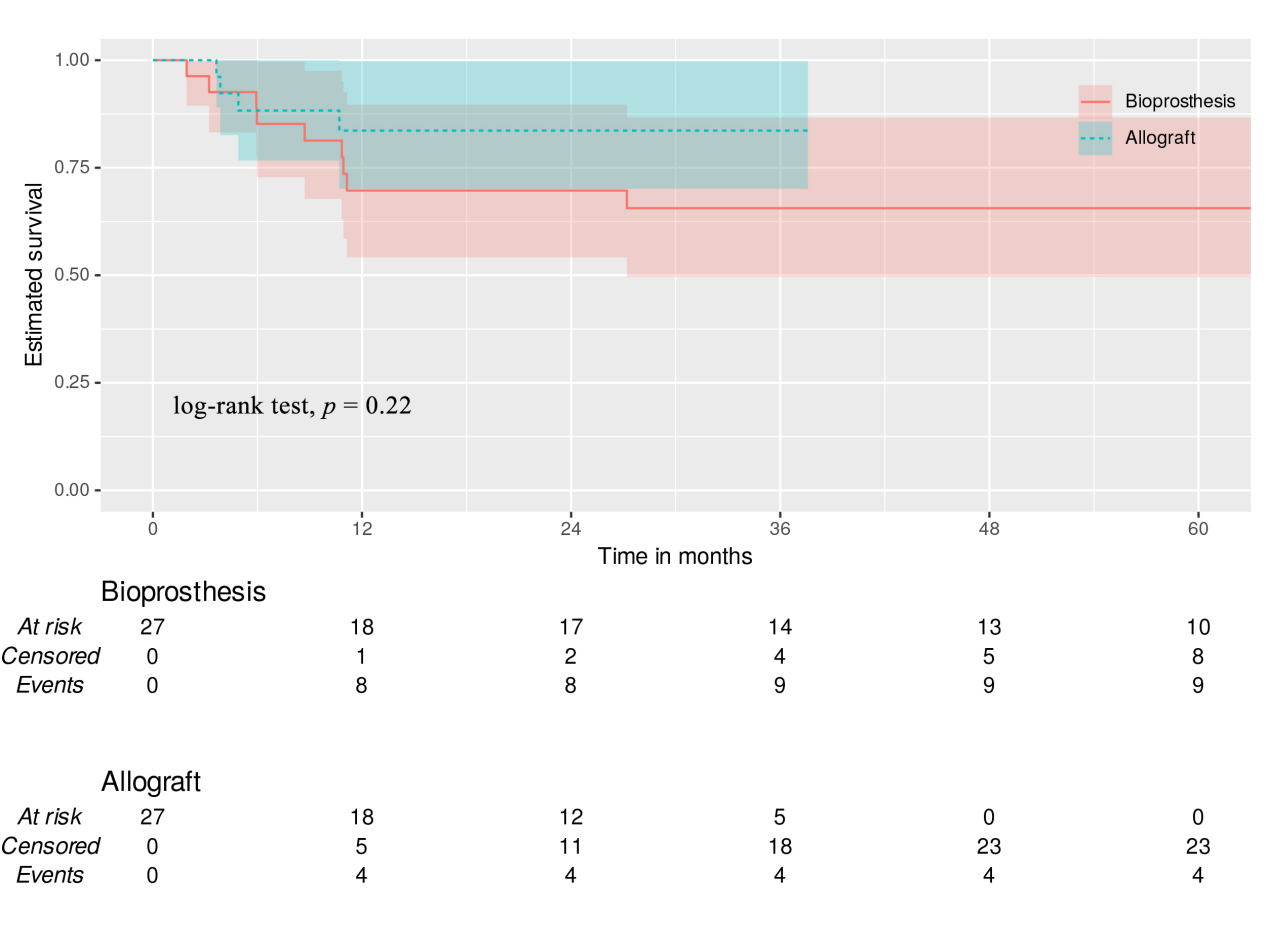
**Endocarditis recurrence**. Green dotted line—allograft with 95% CI. Red line—bioprosthesis with 95% CI.

Most patients experienced IE recurrence during the first postoperative year. In the nine patients in the bioprosthesis group, five presented negative blood cultures while four showed identical microorganisms to the initial episode (suggesting relapse). Two of the four patients in the allograft group had the same microorganism defined at the first episode, while the remaining two had negative blood tests. Most patients in both groups presented with IE recurrence within the first postoperative year. Bioprosthesis TVR and MA demonstrated similar time-to-event freedom from endocarditis recurrence postoperatively (69.7%, 69.7%, 69.7%, 65.6% vs. 83.6%, 83.6%, 83.6%, 83.6% respectively; log-rank test, *p* = 0.22) at 12, 18, 24, and 36 months (Fig. 3).

#### 3.6.4 Pacemaker Implantation

**Fig. 4.**
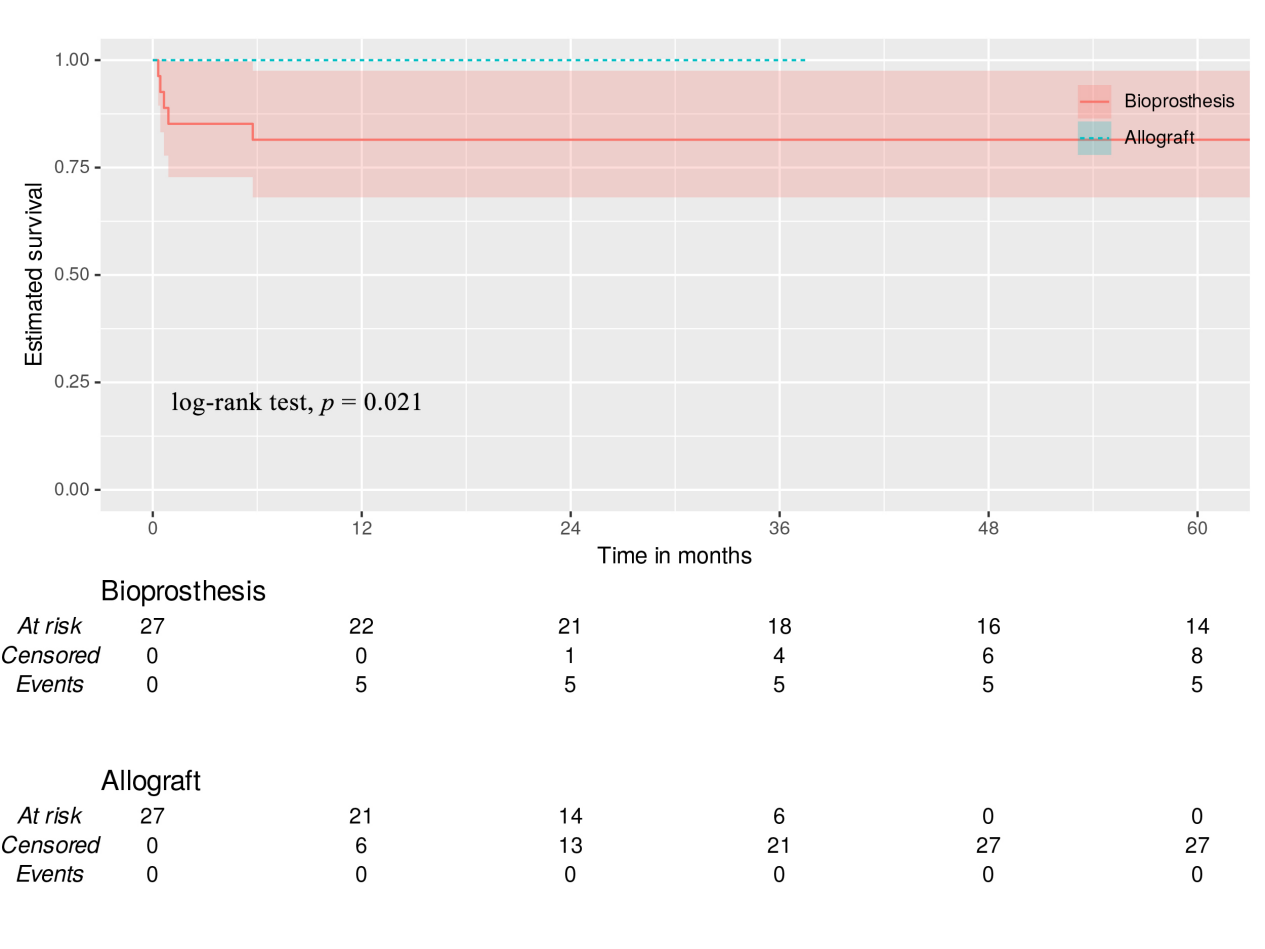
**Permanent pacemaker implantation**. Green dotted line—allograft with 95% CI. Red line—bioprosthesis with 95% CI.

MA demonstrated superior freedom postoperatively from PPI compared to stented bioprosthesis (100% vs. 81% at all time intervals, log-rank test, *p = *0.021) at 12, 18, 24, and 36 months (Fig. 4).

#### 3.6.5 Reoperation

**Fig. 5.**
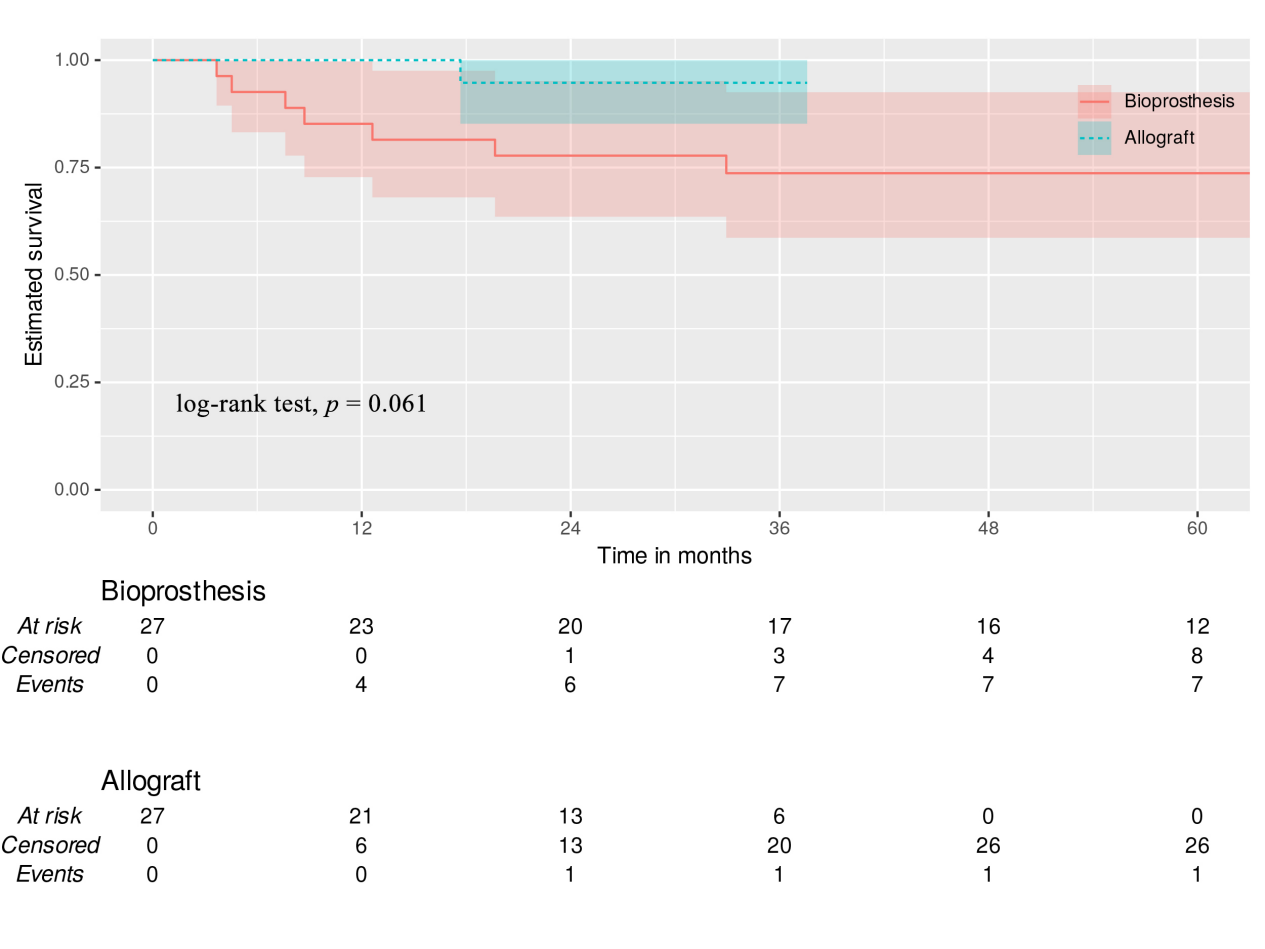
**Reoperation**. Green dotted line—allograft with 95% CI. Red line—bioprosthesis with 95% CI.

Bioprosthesis TVR and MA demonstrated similar time-to-event freedom from reoperation (85.2%, 81.5%, 77.8%, 73.7% vs. 100%, 94.7%, 94.7%, 94.7% respectively; log-rank test, *p* = 0.061) at 12-, 18-, 24-, and 36-months post-operation (Fig. 5). Significantly fewer patients with MA relapse endocarditis required the surgery to be reperformed, since the disease was much more tolerable and could be treated using antibiotics.

## 4. Discussion

Current IE management guidelines provide no consensus on optimal valve substitutes for TV IE when repair is unfeasible [11]. Biological valves are believed to be preferable with regard to management and risks of lifelong anticoagulation and thromboembolic complications, especially in patients with drug addictions [14]. Allogenic tissue valves have also been utilized in patients with TV IE with promising results [7, 8]. The latest study from The Prince Charles Hospital (Australia) demonstrated good long-term survival and freedom from reoperation in patients with IE and Ebstein anomaly, treated using partial tricuspid allograft replacement [15]. However, direct comparison between biological valves and allografts remains lacking. Alternative surgical options have also been proposed, such as percutaneous aspiration and debulking of infective vegetations [16, 17]. Unfortunately, these techniques are unavailable elsewhere and cannot be recommended for all patients. A recent meta-analysis on the comparative outcomes after implanting biological and mechanical valves for TVR showed no significant differences in long-term survival or reoperation rates associated with either type of valve [18]. The authors also found a significant difference in valve failure after five years, with a greater likelihood of failure in mechanical valves. These data are consistent with a previously published systematic review by Cheng *et al*. [19]. Conversely, the new data, derived from a nationwide database study, published by Sohn *et al*. [20] proved survival benefits with mechanical prosthesis, highlighting an ongoing surgical dilemma in prosthetic choice for TVR.

Despite advances in therapeutic and surgical approaches, operative and 30-day mortality following TVR remain significant concerns. Both meta-analyses included studies with relatively high 30-day mortality rates—7.7% (Patlolla *et al*. [21]) and 16.5% (Sohn *et al*. [20]). This study reported zero operative and 30-day mortalities for both biological and allogenic valves, demonstrating improved outcomes in TV IE management. These findings align with the low operative mortality rates reported by Darehzereshki *et al*. [4]. In contrast with other studies, this analysis of mortality is stratified into two subgroups. All-cause mortality showed no significant intergroup difference, while cardiovascular mortality demonstrated statistically superior outcomes with allograft TVR. In other words, the incidence of reperforming surgery and endocarditis relapse in patients with MA in the tricuspid position was not associated with mortality. This could be explained by a more tolerable disease course in patients with an allograft that might imitate native valve endocarditis. This phenomenon likely stems from two key factors: reduced synthetic material compared to stented prostheses (which require pledgets and rigid frames), and superior hemodynamic performance characterized by lower transvalvular pressure gradients. Allogenic leaflets and chords demonstrate greater resistance to calcification and superior long-term mobility. Stented biological valves show predisposition to periannular complications and increased antibiotic resistance. No association was found between bioprosthesis failure and IV drug use status, potentially due to the exclusion of patients classified with active intravenous drug abuse (IVDA) conditions and balanced historical IVDA exposure between groups.

The further issue is the PPI risk, which might reach 16.8% according to Hamandi *et al*. [3]. The prohibited risk of PPI urged the guidelines to recommend prophylactic placement of an epicardial pacing lead during tricuspid surgery [11]. However, this approach may increase the risk of procedure-related complications [11]. This study demonstrates that PPI was implanted in 14.8% of cases after biological TVR, while there was a statistically significant difference with MA, where a zero PPI implantation is highlighted. These data might be considered valuable and comprehensive; PPI was unnecessary in the follow-up period. The surgical technique for allograft implantation may explain these observed differences.

A critical limitation in existing tricuspid valve surgery literature involves the inconsistent application of endpoint definitions, potentially compromising the validity of comparative analyses. The present study implements T-VARC endpoint criteria for comparing valve substitutes regarding early safety and clinical efficacy at particular time points, along with generally applied time-to-event analysis.

Current literature contains limited data regarding IE relapse/recurrence following TV surgery [2]. Meanwhile, existing meta-analyses fail to adequately address freedom from IE—a critical determinant of early prosthetic failure and reoperation. Notably, the allograft group demonstrated no recurrent endocarditis cases among patients with documented IVDA history, indicating a complete absence of relapse in this high-risk subgroup. This study shows that endocarditis recurrence in patients with stented bioprosthesis is associated with reoperation, since half of the patients with recurrent endocarditis in the bioprosthesis group underwent redo surgery. Only one patient with an allograft experienced significant valve failure and was scheduled for the reperformance of surgery. Two-year clinical efficacy assessments using T-VARC criteria demonstrated superior outcomes with mitral allografts. Time-to-event survival (freedom from surgery reconduction and recurrent endocarditis) demonstrated similar survival in both groups. The difference between T-VARC clinical efficacy and Kaplan–Meier survival could be explained by the different methods used in the statistical analyses. The comparable baseline characteristics between groups suggest that similar time-to-event outcomes, including endocarditis recurrence rates, may reflect genuine clinical equipoise rather than statistical artifacts.

A notable gap in the existing literature concerns the hemodynamic evaluation of valve substitutes. Meanwhile, this study revealed that only 12 (44.4%) patients with stented bioprosthesis exhibited a proper transtricuspid mean gradient, conforming to the criteria of T-VARC consensus, while allografts had a significantly better hemodynamic profile at discharge from the hospital; these results were sustained at the 30-day and 2-year time points.

## 5. Conclusions

This study represents the first comparative analysis of stented biological valves and mitral allografts in the surgical treatment of TV IE. Both valve substitutes provide satisfactory early results, compared to previous studies. Mitral allograft demonstrated superior results over stented biological prosthesis in terms of cardiovascular mortality, permanent pacemaker implantation, and hemodynamic profile. The application of a contemporary endpoint definition at 2 years after surgery also showed the benefits of the allograft valve in terms of recurrent endocarditis, reoperation, cardiac hospitalization, and the self-reported outcomes of patients. A time-to-event analysis did not demonstrate a significant difference in all-cause mortality, recurrence of IE, and surgery reperformance. A mitral allograft may be an alternative valve substitute for TV replacement in patients with infective endocarditis, with superior outcomes.

## Availability of Data and Materials

All data reported in this paper might be shared by the lead contact upon request.

## Supplementary Material

Supplementary material associated with this article can be found, in the online version, at https://doi.org/10.31083/RCM37204.
