# Supplementary material for: Stented Biological Prosthesis Versus Mitral Allograft in Surgical Treatment of Tricuspid Valve Infective Endocarditis
Source: Rev Cardiovasc Med. 2025 Jul 8;26(7):37204. doi: 10.31083/RCM37204 (PMC12326450; doi:10.31083/RCM37204)
Supplement: Supplementary file 1 [file 2153-8174-26-7-37204-s1.zip › Supplementary Table 1.docx]

Supplementary Table 1. Operative details

|  | **Bioprosthesis** | **Allograft** | ***p*** |
| --- | --- | --- | --- |
| Total N (%) | 27 (50.0) | 27 (50.0) |  |
| CCT, min | 58.0 (50.5 to 70.5) | 118.0 (104.5 to 132.5) | **<0.001** |
| CPB, min | 85.0 (69.5 to 104.0) | 143.0 (122.5 to 155.5) | **<0.001** |
| Ventilation time, h | 7.0 (6.0 to 10.5) | 7.0 (5.0 to 8.5) | 0.394 |
| Drainage output (first 24 hours), ml | 300.0 (200.0 to 355.0) | 300.0 (250.0 to 450.0) | 0.449 |
| Red cell transfusion, ml | 700.0 (230.0 to 989.5) | 620.0 (0.0 to 920.0) | 0.587 |
| Fresh frozen plasma, ml | 1020.0 (585.0 to 1115.0) | 720.0 (0.0 to 855.0) | **0.022** |
| PLT transfusion, n(%) |  |  | 1.000 |
| 330 ml | 1 (3.7) | 0 (0.0) | 1.000 |
| 340 ml | 0 (0.0) | 1 (3.7) |  |
| 360 ml | 0 (0.0) | 1 (3.7) |  |
| Cryoprecipitate transfusion, n(%) | 1 (3.7) | 1 (3.7) | 1.000 |

Table footnote: Data are expressed as a number (n, (%)) or Mediana (Interquartile range). CCT, cross clamp time; CPB, cardiopulmonary bypass time; PLT, platelets; IQR, interquartile range;
