# Supplementary material for: Stented Biological Prosthesis Versus Mitral Allograft in Surgical Treatment of Tricuspid Valve Infective Endocarditis
Source: Rev Cardiovasc Med. 2025 Jul 8;26(7):37204. doi: 10.31083/RCM37204 (PMC12326450; doi:10.31083/RCM37204)
Supplement: Supplementary file 1 [file 2153-8174-26-7-37204-s1.zip › Supplementary Table 2.docx]

Supplementary Table 2. Preoperative and postoperative echocardiographic data

|  | **Bioprosthesis** | **Allograft** | ***p*** |
| --- | --- | --- | --- |
| Total N (%) | 27 (50.0) | 27 (50.0) |  |
| LV EF before, % | 64.0 (60.5 to 66.0) | 65.0 (62.0 to 67.0) | 0.353 |
| LV EF after, % | 63.0 (59.5 to 65.0) | 64.0 (60.5 to 66.5) | 0.344 |
| RV size before, mm | 34.0 (31.0 to 36.0) | 35.0 (31.0 to 37.5) | 0.405 |
| RV size after, mm | 29.0 (27.0 to 31.0) | 30.0 (27.5 to 31.5) | 0.676 |
| TV annulus, mm | 35.0 (31.5 to 40.5) | 36.0 (33.0 to 41.0) | 0.362 |
| RA size before, mm | 56.0 (50.5 to 60.5) | 55.0 (49.5 to 61.0) | 0.897 |
| RA size after, mm | 48.0 (45.5 to 49.5) | 47.0 (44.0 to 52.0) | 0.958 |
| PAP before, mmHg | 50.0 (40.0 to 55.0) | 40.0 (38.0 to 50.0) | 0.103 |
| PAP after, mmHg | 35.0 (30.0 to 38.0) | 30.0 (30.0 to 33.5) | **0.023** |
| TV VC before, cm | 1.0 (0.7 to 1.4) | 0.8 (0.7 to 1.1) | 0.343 |
| TV VC after, cm | 0.1 (0.1 to 0.2) | 0.1 (0.1 to 0.2) | 0.947 |
| Peak TV PG after, mmHg | 9.2 (6.5 to 12.0) | 6.0 (4.5 to 7.5) | **0.001** |
| Mean TV PG after, mmHg | 4.0 (3.2 to 6.0) | 3.0 (2.0 to 4.0) | **0.001** |

Table footnote: Data are expressed as a number (n, (%)) or Mediana (Interquartile range). LV EF - left ventricle ejection fraction; RV, right ventricle; RA, right atrium; TV - tricuspid valve; PAP, pulmonary artery pressure; TV VC, tricuspid valve vena contracta; TV PG, tricuspid valve pressure gradient; IQR, interquartile range;
